# A 900 μm^2^ BiCMOS Temperature Sensor for Dynamic Thermal Management

**DOI:** 10.3390/s20133725

**Published:** 2020-07-03

**Authors:** Hernán Aparicio, Pablo Ituero

**Affiliations:** Departamento de Ingeniería Electrónica, IPTC, ETSI Telecomunicación, Universidad Politécnica de Madrid, Avda. Complutense 30, 28040 Madrid, Spain; hernan@die.upm.es

**Keywords:** power supply, PVT variations, variability-aware design

## Abstract

The extreme miniaturization of electronic technologies has turned varying and unpredictable temperatures into a first-class concern for high performance processors which mitigate the problem employing dynamic thermal managements control systems. In order to monitor the thermal profile of the chip, these systems require a collection of on-chip temperature sensors with strict demands in terms of area and power overhead. This paper introduces a sensor topology specially tailored for these requirements. Targeting the 40 nm CMOS technology node, the proposed sensor uses both bipolar and CMOS transistors, benefiting from the stable thermal characteristics of the former and the compactness and speed of the latter. The sensor has been fully characterized through extensive post-layout simulations for a temperature range of $0{\;}^{\circ }C$ to $100{\;}^{\circ }C$, achieving a maximum error of ±0.9${\;}^{\circ }C$/ considering 3σ yield and a resolution of 0.5${\;}^{\circ }C$. The area—900 μm${}^{2}$, energy per conversion—1.06 nJ, and sampling period—2 μs, are very competitive compared to previous works in the literature.

## 1. Introduction

Nowadays, competition among integrated circuit (IC) companies to offer more features involves a great effort to make the design of their chips very robust to process, voltage, and temperature (PVT) variations. The main concerns of the industry about PVT variations are related to the loss of performance, reliability, and the cost of low fabrication yield.

Focusing on the temperature, the advanced chips used in mobile phones, computers, and other devices that operate in real-time are made by billions of transistors, which means an extremely high density of components inside modern CPUs and GPUs. The transistor density associated with high operation frequencies implies elevated power densities which in turn are translated into high on-chip temperatures.

Varying unpredictable temperatures negatively impact the normal behavior of ICs in many ways: Alterations of the device’s nominal responses that produce delays uncertainties and analog mismatches; exacerbation of the aging processes which is translated into reduced lifetimes; increased static power consumption; reliability issues produced by thermal noise; interconnection expansions that cause mechanical stress and affect fundamental signals such as the power supply or the clocking network. In this scenario, on-chip thermal monitoring has become more and more employed and plays a key role in assisting the dynamic thermal management (DTM) system. In particular, a set of distributed on-chip temperature sensors provides real-time information about the thermal profile of the chip.

Being part of a monitoring system separated from the main IC functionality entails that the area and power overhead of on-chip temperature sensors must be kept as low as possible. The accuracy of the sensors must be high enough to fulfill the requirements of the DTM policies, albeit this restriction is not very tight compared to other applications. This poses several design challenges that separate this type of temperature sensors from those specifically tailored to obtain very high accuracy, leaving the area and power consumption as secondary constraints. Digital outputs (smart sensing), operation without the need for an external reference, and no or straightforward calibration processes are other desirable characteristics of on-chip temperature sensors specifically designed for DTM [1].

As far as the temperature sensors designed to achieve a high accuracy are concerned, devices of one type stood out as the one with better robustness against variability: bipolar junction transistors (BJTs) [2,3,4,5,6]. The emitter-base voltage of a BJT presents a linear and stable response to temperature because of the thermal voltage. For this reason, the bipolar transistor is typically used to provide a voltage proportional-to-absolute temperature (VPTAT). This voltage can be directly digitized by employing an analog-to-digital converter (ADC), or it can be converted into a thermal-dependent current source that produces a periodic signal whose frequency is digitized by employing a frequency-to-digital converter (FDC). Either way requires complex circuitry that is translated into a big area overhead.

Additionally, while displaying good predictability, temperature sensors based on resistors [7,8,9,10,11] such as BJT sensors have high linearity and accuracy, but on the other hand, consume more area and add additional noise into the circuit. New technology nodes that work with supply voltages under 1 V have made resistance temperature sensors an alternative to BJT sensors because the base-emitter voltage, $V_{be}$, of the BJTs is around 0.7, which is very close to the power supply. It is worth mentioning that the resistance has a higher temperature sensitivity which makes it possible to relax the requirements of the analog blocks used to convert the temperature into digital values.

During the last decade, there have been numerous proposals for temperature sensors mainly oriented towards the needs of DTM systems [1,12,13,14,15,16]. They usually have a common feature which is avoiding the use of ADCs to save area and power consumption; instead, they employ a time-to-digital converter (TDC) or a (FDC) to perform the digitization. The sensing mechanism of this type of architecture can be illustrated by an inverted-based ring oscillator. The delay of each inverter in the oscillator has an almost-linear dependence on the temperature and so does the oscillation period. A digital counter is used as an FDC to perform the digitization. This type of architecture is small, easy to integrate, and requires little power consumption; however, providing high accuracy values is challenging as it is very prone to process variations.

In this work, we propose a novel smart temperature sensor architecture that tries to join the benefits of the BJT’s linear response with the simplicity of DTM-oriented proposals. In particular, we propose a ring-oscillator structure that employs BJTs as pull-up devices, so that the thermal dependency of the oscillation frequency comes from that of the base-emitter voltage. Besides, no external reference and no conventional voltage-to-current converter are required. Employing BJTs is translated into very low process variation, and the digitization strategy makes the sensor have a small area and less power consumption when compared with previous works. The sensor was designed using a 40 nm CMOS process, and all process corners were tested in a wide range of temperatures ($0{\;}^{\circ }C$–$100{\;}^{\circ }C$). The proposed sensor exhibits the following features:

- It covers a temperature range between $0{\;}^{\circ }C$ and $100{\;}^{\circ }C$.
- Resolution of 0.5 ${\;}^{\circ }C$.
- Inaccuracy of ±0.9 ${\;}^{\circ }C$/ considering both process and Monte Carlo analysis.
- Per measurement, 1.06 nJ of energy consumption.
- Area: 900 μm${}^{2}$.

The rest of this paper is organized as follows. Section 2 describes the proposed sensor architecture and develops the analytic equations that describe its temperature transfer function. Section 3 shows the post-layout simulations and compares its results with previous works. At last, a conclusion is given in Section 4.

## 2. Architecture of the Proposed Temperature Sensor and Analysis

**Figure 1:**
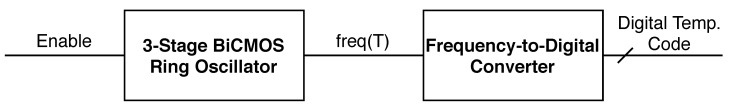
Temperature sensor based on CMOS.

The temperature sensor architecture of this work is based on Figure 1, and it is composed of a temperature-sensitive ring oscillator and a frequency-to-digital converter. The ring oscillator (RO) is formed by three BiCMOS inverters and has an oscillation frequency dependent on the temperature. A counter converts the oscillator frequency into a digital output. For this kind of architecture, the total power consumption is dominated by the ring oscillator, which is the sensing element. A new BiCMOS inverter was developed to have an output delay dependent on the temperature making this architecture to be based both on BJTs and CMOS.

To measure the temperature, the ring oscillator is activated during a fixed time period, and the counter counts the number of oscillations. The temperature sensor is assembled by a ring oscillator with only three BiCMOS inverters and one counter which entails a reduced power consumption and a very compact design. In the next section, we present the BiCMOS inverter and explain how the ring oscillator is built.

### 2.1. BiCMOS Inverter and Ring Oscillator

**Figure 2:**
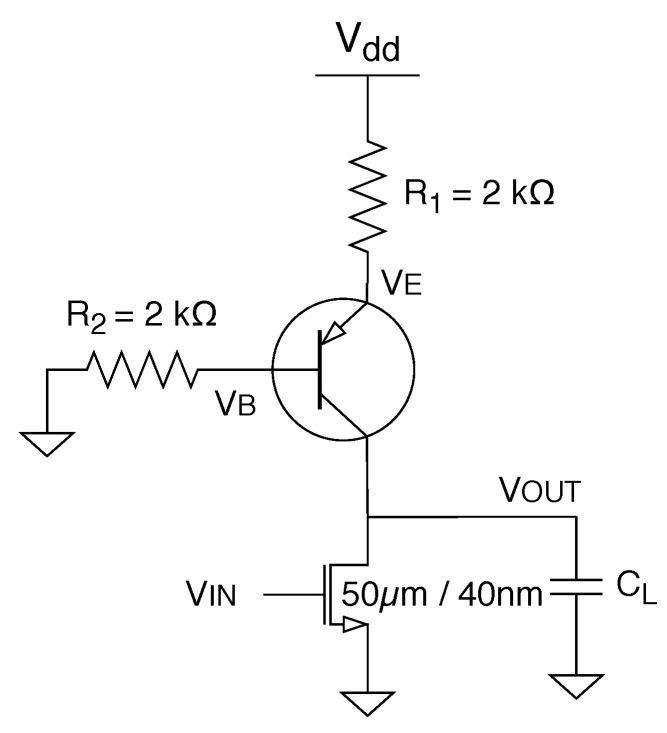
BiCMOS inverter.

Figure 2 shows the BiCMOS inverter. The inverter is constituted by a PNP BJT, two resistances independent on temperature, and an NMOS transistor. When the input of the circuit is 0 the NMOS transistor is cut off, and for the steady state, the PNP BJT operates in saturation mode wherein the emitter-base junction and the collector-base junction are forward biased. The steady state value of the output is dependent on the temperature; however, it will not play an important role in this temperature sensor. When the input is higher than the NMOS threshold voltage ($V_{th}$), both the pull-up and the pull-down networks will be active; however, making the width of the NMOS transistor big enough forces the output close to zero.

Saturated bipolar digital circuits generally are no longer the technology of choice in digital system design because their speed of operation is severely limited by the relatively smaller currents and longer time delays required to turn off a saturated transistor [17]; however, this supposes an advantage in this case, as we can construct a ring-oscillator with a manageable frequency with very few stages; this is translated into a more compact design and less switching nodes, and thus less power consumption.

**Figure 3:**
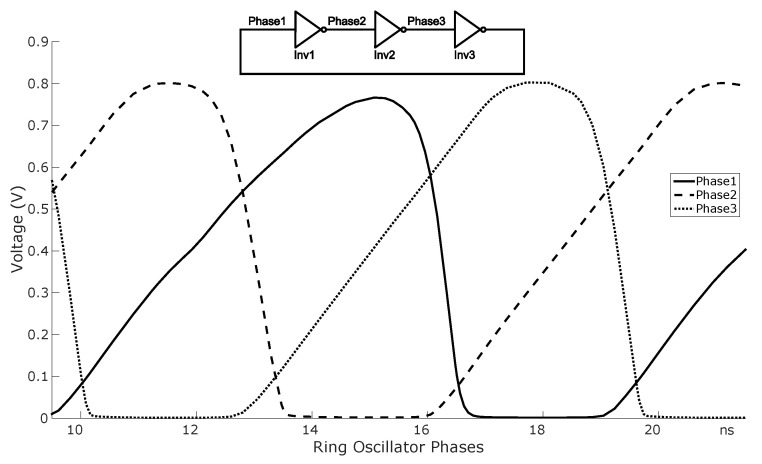
BiCMOS Inverter.

Turning now to the operation of the ring oscillator, Figure 3 shows the transient voltages of the three nodes of the oscillator, Phase1, Phase2, and Phase3. The waveforms are easier to understand if we focus on the transient behavior of one of the inverters. Let us suppose we depart from a state in which $V_{O}\approx 0$ and $V_{IN}$ change from above $V_{th}$ to below $V_{th}$. The NMOS transistor will cut off, and the output node will start charging up through the collector current, $I_{C}$, of the BJT. Whenever the output surpasses the next stage NMOS $V_{th}$, the ripple will propagate to the next stage. Now we consider the moment when $V_{IN}$ surpasses the NMOS $V_{th}$, at that time the output node will discharge through this transistor. The sizing of the NMOS transistor has been carefully chosen so that the discharge time (the time it takes to discharge from the previous high value to $V_{th}$) is very small in comparison to the charge time (from 0 V to $V_{th}$); in particular, the aspect ratio of the NMOS was designed to be equal to 1250.

Under these conditions, the 3-stage ring oscillator period is three times the charging time of each node from 0 V to $V_{th}$. This charging time depends on $I_{C}$, the node capacitance, and $V_{th}$. In the next sections we extract the analytical model of the thermal dependency of $I_{C}$ and provide a closed-form expression of the frequency of the ring oscillator as a function of the temperature.

### 2.2. Collector Current Analysis

**Figure 4:**
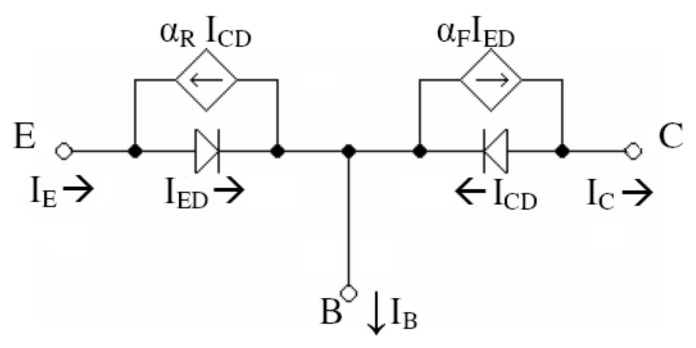
Ebers–Moll model for the PNP transistor.

We want to express $I_{C}$ as a function of the temperature. For that, we substitute the PNP transistor by its Ebers–Moll model, as shown in Figure 4.

**Figure 5:**
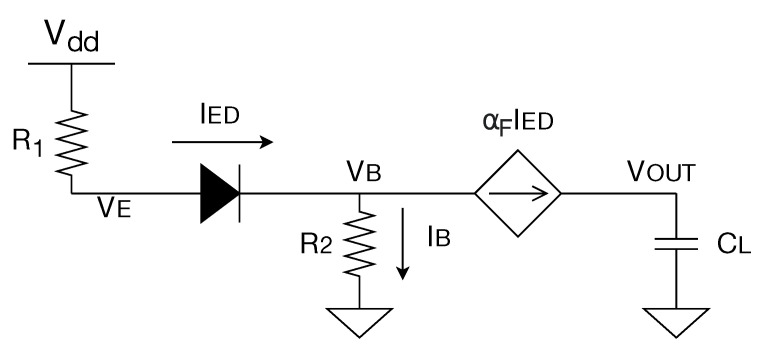
Equivalent circuit for the proposed sensor when $V_{IN}=0$.

In our analysis, we establish that $V_{IN}=0$ and that $V_{OUT}$ charges from 0 V to $V_{th}$; if we consider $V_{B}$ to be very close to 0, we can conclude that the collector-base diode will not be active. Figure 5 shows the equivalent circuit. With these assumptions we can develop the analytic equations of the proposed circuit:

- PNP currents
  (1) $$I_{E}=I_{B}+I_{C}$$
- $I_{C}$ current
  (2) $$I_{C}=\alpha_{F}I_{E}$$
- $I_{B}$ current
  (3) $$I_{B}=\left(1-\alpha_{F}\right)I_{E}$$
- $V_{E}$ voltage
  (4) $$V_{1}=V_{DD}-I_{E}R_{1}$$
- $V_{B}$ voltage
  (5) $$V_{B}=I_{B}R_{2}=\left(1-\alpha_{F}\right)I_{E}R_{2}$$
- Diode equation of the emitter-base junction:
  (6) $$I_{E}=I_{ED}=I_{S}\left(e^{\left(V_{E}-V_{B}\right)/V_{T}}-1\right)\approx I_{S}e^{\left(V_{E}-V_{B}\right)/V_{T}}$$

In this equation, $I_{S}$ is the reverse saturation current of the base–emitter diode and $V_{T}$ is the thermal voltage. Both parameters have a strong thermal dependence, as will be explained at the end of this analysis. Now, substituting (4) and (5) in (6) and considering $R_{3}=R_{1}+R_{2}\left(1-\alpha_{F}\right)$ we obtain:

(7) $$I_{E}=I_{S}\left(e^{\left(V_{DD}-I_{E}R_{3}\right)/V_{T}}\right)$$

Employing the W Lambert function, we obtain the following expression of $I_{E}$:

(8) $$I_{E}=\frac{V_{T}}{R_{3}}W\left(\frac{I_{S}R_{3}}{V_{T}}e^{\frac{V_{DD}}{V_{T}}}\right)$$

With the first-order approximation of the W Lambert function we have the following expression for $I_{E}$:

(9) $$I_{E}\approx \frac{V_{T}}{R_{3}}ln\left(\frac{I_{S}R_{3}}{V_{T}}\right)+\frac{V_{DD}}{R_{3}}$$

So, for $I_{C}$, we have

(10) $$I_{C}=\alpha_{F}I_{E}=\frac{\alpha_{F}V_{T}}{R_{3}}ln\left(\frac{I_{S}R_{3}}{V_{T}}\right)+\frac{\alpha_{F}V_{DD}}{R_{3}}$$

Note that this expression matches that of a current source independent on the output voltage. Focusing now on the thermal analysis, let us recall the temperature dependencies of the variables that take part in the circuit behavior.

Concerning $I_{S}$, it has a the following dependence on the temperature:

(11) $$I_{S}\left(T\right)=\frac{A_{E}qD_{n}B}{N_{A}W_{b}}T^{3}e^{-\frac{E_{G}}{kT}}=\xi_{1}T^{3}e^{-\frac{E_{G}}{kT}}$$

where $A_{E}$ is the cross-sectional area of the base-emitter junction, *q* is the magnitude of the electron charge $=1.60\times 10^{-19}$ C, $D_{n}$ is the electron diffusivity in the base, $W_{b}$ is the effective width of the base, $N_{A}$ is the doping concentration in the base, *B* is a material-dependent parameter $=5.4\times 10^{31}$ for silicon, $E_{G}$ is a parameter known as the bandgap energy =1.12 electron volts (eV) for silicon, and *k* is Boltzmann’s constant $=8.62\times 10^{-5}$ eV/K.

The thermal voltage, $V_{T}$, has a simpler thermal dependence:

(12) $$V_{T}\left(T\right)=\frac{kT}{q}$$

$R_{1}$ and $R_{2}$, and thus $R_{3}$, have a negligible dependency on temperature because the technology used for this design provides non-silicide P + poly-resistors that are robust against temperature variations.

Substituting (11) and (12) in (10) we have:

(13) $$\begin{array}{cc} I_{C}\left(T\right) & =\alpha_{F}I_{E}=\frac{\alpha_{F}kT}{qR_{3}}ln\left[\frac{T^{3}e^{-\frac{E_{G}}{kT}}\xi_{1}qR_{3}}{kT}\right]+\frac{\alpha_{F}V_{DD}}{R_{3}}=\\ \; & =\frac{\alpha_{F}kT}{qR_{3}}\left[ln\left(\frac{\xi_{1}qR_{3}}{k}\right)+ln\left(T^{2}\right)-\frac{E_{G}}{kT}\right]+\frac{\alpha_{F}V_{DD}}{R_{3}}=\\ \; & =\frac{\alpha_{F}}{qR_{3}}\left[E_{G}+qV_{DD}+Tk\left[ln\left(\frac{\xi_{1}qR_{3}}{k}\right)+ln\left(T^{2}\right)\right]\right] \end{array}$$

The term $ln(T^{2})$ has very little variation in the temperature range of operation, so we can approximate it by its value in the middle of the range; i.e., $ln\left(T^{2}\right)\approx ln\left(T_{middle}^{2}\right)$. With this approximation, we can express $I_{C}$ as a linear function of the temperature.

(14) $$I_{C}\left(T\right)=\zeta_{1}+T\zeta_{2}$$

considering

(15) $$\zeta_{1}=\frac{\alpha_{F}}{qR_{3}}\left(E_{G}+qV_{DD}\right)$$

(16) $$\zeta_{2}=\frac{\alpha_{F}k}{qR_{3}}\left[ln\left(\frac{\xi_{1}qR_{3}}{k}\right)+ln\left(T_{middle}^{2}\right)\right]$$

The bottom line of this analysis is that the BJT behaves as a current source whose value increases linearly with the temperature.

### 2.3. Ring Oscillator Frequency Thermal Dependence

Now that $I_{C}$ has been shown to act as a current source, we can extract the expression of the charging time of each stage in the ring oscillator:

(17) $$\int_{t=0}^{t=t_{r}}I_{C}\left(t\right)\cdot dt=C_{L}V_{th}$$

Thus,

(18) $$t_{r}=\frac{C_{L}V_{th}}{I_{C}}$$

The expression of the 3-stage oscillator frequency is the following:

(19) $$f=\frac{I_{C}}{3C_{L}V_{th}}$$

Concerning the thermal dependence, $I_{C}$ has already been analyzed and $C_{L}$ is mostly composed of the gate capacitance of the next stage NMOS transistor, which is hardly affected by temperature changes. The remaining parameter, $V_{th}$, has a linear function of temperature and can be modeled as showen in [18]:

(20) $$V_{TH}\left(T\right)=V_{TH}\left(T_{NOM}\right)+\left(K_{V}+\frac{K_{CL}}{L}+K_{BS}V_{BS}\right)\left(\frac{T}{T_{NOM}}-1\right)$$

where $V_{TH}\left(T_{NOM}\right)$ is the threshold voltage at a nominal temperature, $K_{V}$ is the temperature coefficient of threshold voltage, $K_{CL}$ is the channel length ratio of the temperature dependence of the threshold voltage, and $K_{BS}$ is the coefficient of bulk-bias temperature dependence of the threshold voltage. As the model shows, as the temperature rises, the absolute value of the threshold voltage decreases. To simplify the notation, let us group all the thermal independent parameters:

(21) $$V_{TH}\left(T\right)=\zeta_{3}+T\zeta_{4}$$

considering

(22) $$\zeta_{3}=V_{TH}\left(T_{NOM}\right)-K_{V}-\frac{K_{CL}}{L}-K_{BS}V_{BS}$$

(23) $$\zeta_{4}=\frac{1}{T_{NOM}}\left(K_{V}+\frac{K_{CL}}{L}+K_{BS}V_{BS}\right)$$

At this point we are able to provide a closed-form expression that relates the frequency of the 3-stage ring oscillator and the temperature:

(24) $$f\left(T\right)=\frac{I_{C}}{3C_{L}V_{th}}=\frac{\zeta_{1}+T\zeta_{2}}{3C_{L}\left(\zeta_{3}+T\zeta_{4}\right)}$$

As the temperature rises, $I_{C}$ grows and $V_{th}$ diminishes, so both factors contribute to increase the frequency. Although Equation (24) is not a linear function and some simplifications have been taken, experimental results will show that for the application range of temperatures, this structure achieves linearity and accuracy levels comparable to those of other works in the literature.

### 2.4. Frequency-to-Digital Converter

**Figure 6:**
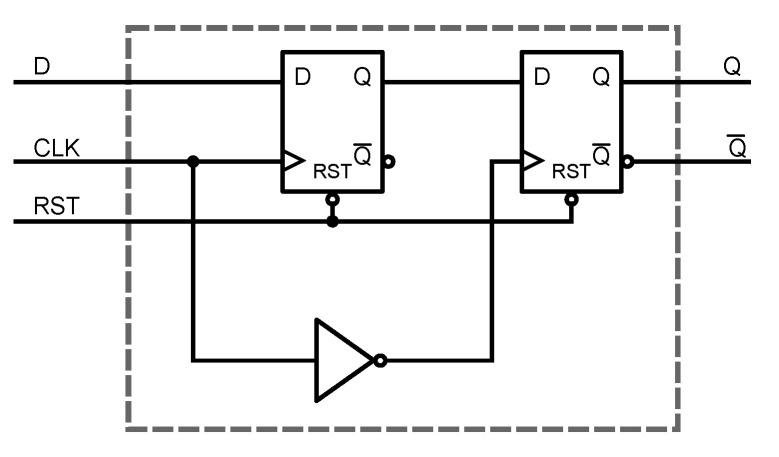
Master–slave D flip-flop.

The counter designed to convert the frequency from the ring oscillator to a digital value was based on the traditional master–slave D flip-flop, shown in Figure 6. The 12-bit counter is formed by 12 D flip-flop cells connected in series; the temperature-dependent oscillation feeds the clock; and an external fixed-length pulse enables and resets the count. The resolution of the sensor is fixed by both the size in bits of the counter and the length of the external pulse.

## 3. Post-Layout Simulation Results and Comparison with Previous Works

This section presents the simulation results of the proposed temperature sensor and compares them with previous works. The sensor was designed and validated in a 40 nm CMOS technology-powered at 1.1 V. The results come from transient simulations that cover temperatures from $0{\;}^{\circ }C$ to $100{\;}^{\circ }C$. All simulations were done after post-layout routing taking into account the parasitics extraction. Both wost-corner and Monte Carlo analysis were run to obtain the results.

**Figure 7:**
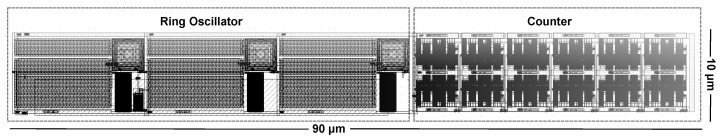
Layout of the proposed temperature sensor.

The area of the sensor is 900 μm${}^{2}$, Figure 7 shows the layout of the design. As shown, the floorplan is well divided between the ring oscillator and the digitization counter. The simplicity of the structure makes the design specially compact.

**Figure 8:**
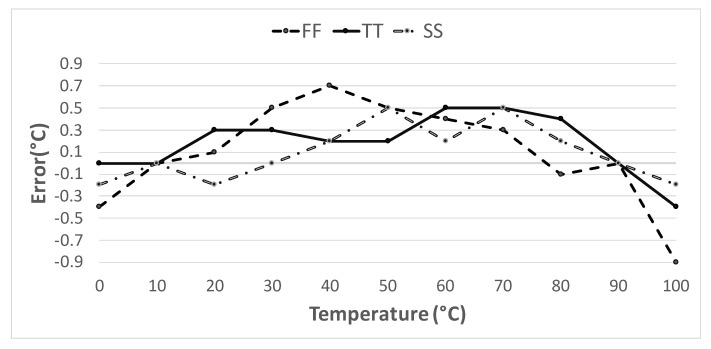
Temperature error considering all process corners.

The sensor accuracy, ±0.9${\;}^{\circ }C$ was established by means of both Monte Carlo and corners simulations in a range from $0{\;}^{\circ }C$ to $100{\;}^{\circ }C$, taking values every $10{\;}^{\circ }C$, and performing a 2-point calibration at $10{\;}^{\circ }C$ and $90{\;}^{\circ }C$. The results of the corner analysis can be seen in Figure 8.

**Figure 9:**
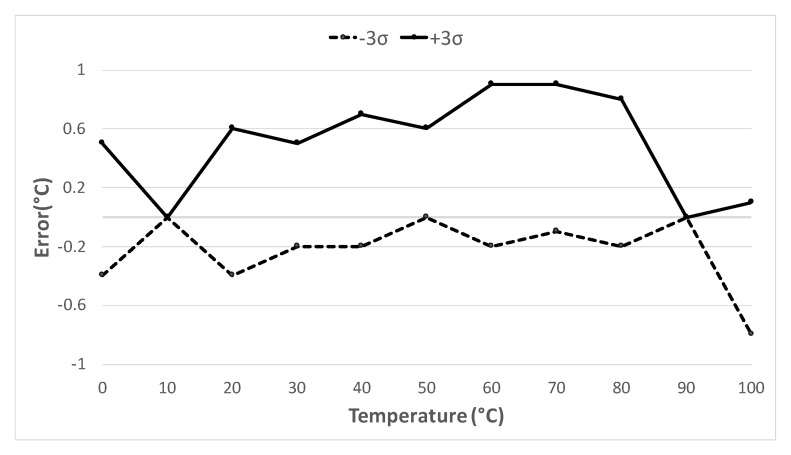
Monte Carlo boundaries (+3σ and −3σ).

Figure 9 shows the temperature error boundaries of the Monte Carlo post-layout simulations. One hundred samples were taken for each temperature point. As shown, the results are consistent with the corner analysis.

The resolution and the sampling period of the sensor are fixed by the size of the counter that performs the digitization. A bigger counter can provide with higher resolutions, but also needs bigger sampling periods. The temperature sensor was designed to match the requirements of DTM systems that need a big collection of on-chip monitors with the minimum overhead. Therefore, in accordance to the accuracy of the sensor and to minimize the counter area and power consumption, the resolution was fixed around $0.5{\;}^{\circ }C$ and the sampling period to 2 μs. With this sampling period, the average value of the energy per conversion is 1.06 nJ.

**Figure 10:**
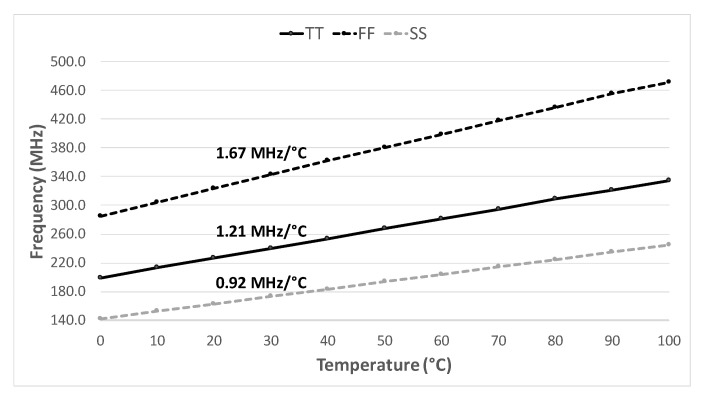
Frequency sensitivity over temperature and process corners.

For the $0{\;}^{\circ }C$ to $100{\;}^{\circ }C$ temperature range considering all process corners, the oscillation frequency changes at a rate from 0.92 Hz/°C to 1.67 MHz/°C showed in Figure 10. As can be seen, the resolution is higher for the FF corner and lower for the SS corner.

Selecting a short collection of previous works for comparison in the field of temperature sensors is complicated due to the vast existing literature. Prof. K.A.A. Makinwa keeps an excellent online survey of published sensors [19] that has been used to choose those that present similar characteristics and target DTM (BJT and CMOS) or that present a singular feature, such as extremely high accuracy [4], or were fabricated in a very small node [3].

Table 1 summarizes the main characteristics of the temperature sensor and compares them with the selected previous works. Area information is provided by all sensors; as shown, the proposed work has the smallest figure of merit in this row. Some previous works [3,5,13,15] have been fabricated in smaller CMOS technology nodes but occupy more area. Additionally, the sampling period (Ts) in our proposal is very reduced, 2 μs, which is translated into a very low value for the energy per conversion, 1.06 nJ. As can be seen, our temperature sensor is characterized by a robust behavior when considering process and parasitics extractions, which are very significant in the 40 nm technology node. The size of the temperature range between $0{\;}^{\circ }C$ to $100{\;}^{\circ }C$ is also comparable to the rest of the previous works.

## 4. Conclusions

This paper has presented a temperature sensor fabricated in the 40 nm technology node and specially tailored for the requirements of DTM systems. We have tried to reduce as much as possible both the amount of hardware resources and the energy budget. The seed idea was the BiCMOS inverter composed by a BJT in the pull-up network and an n-MOS transistor in the pull-down network. The BJT makes the rise time of the inverter slow, temperature dependent, and especially invariant against process and voltage variations. This long rise time allowed us to build a ring oscillator with just three stages that yields a frequency-varying signal that can be digitized by means of a simple counter. An external pulse activates the oscillator and sets the counter to count the number of pulses during a fixed time lapse. No external reference is needed to operate the sensor.

The temperature sensor features have been validated through post-layout simulations that cover a temperature range of $0{\;}^{\circ }C$ to $100{\;}^{\circ }C$ and all process corners. The resulting design is more compact, 900 μm${}^{2}$, and less energy hungry, 1.06 nJ/operation, than comparable previous works in the literature. Additionally, the sampling period, 2 μs, stands out as a very interesting feature. The sensor has a resolution of 0.5${\;}^{\circ }C$, which is dependent on the fixed length of the external signal that controls the counter. For the accuracy figures, we have performed a 2-point calibration at $10{\;}^{\circ }C$ and $90{\;}^{\circ }C$, obtaining a maximum error of ±0.9${\;}^{\circ }C$/ considering 3σ yield.

## Figures and Tables

**Table 1:** Comparison with other voltage monitors described in the literature.

	This Work	[3]	[4]	[5]	[6]	[12]	[13]	[14]	[15]
Sensor Type	BiCMOS	BJT	BJT	BJT	BJT	CMOS	CMOS	CMOS	CMOS
Technology (nm)	40	20	160	22	65	65	25	180	28
Temp Range (${}^{\circ }C$)	0∼100	−25∼125	−70∼125	−30∼120	−10∼110	0∼100	20∼95	−20∼80	−5∼85
Vdd Supply (V)	1.1	1.8	1.5 ∼ 2	1	1.3	1	1.1	1.8	0.9
Resolution (mK)	500	400	15	580	130	300	50	90	760
Ts (ms)	0.002	0.16	5	0.03	4.1	0.022	0.142	800	0.036
Trim	2	1	1	1	2	2	1	2	1
Inaccuracy (${}^{\circ }C$)	±0.9	±2.5	±0.05	±1.07	±1.35	±0.9	±2	±1	±1.35
Area (mm${}^{2}$)	0.0009	0.018	0.16	0.0043	0.0030	0.004	0.02	0.089	0.001
Power (μW)	530	1100	6.9	50	111.8	154	9	0.8	56
Energy (nJ)	1.06	180	35	1.6	460	3.4	1.3	660	2
FOM [19]	0.27	28	0.0078	0.54	7.7	0.3	0.0032	5.3	1.2
